# The effect of crude guava leaf tannins on motility, viability, and intact plasma membrane of stored spermatozoa of Etawa crossbred goats

**DOI:** 10.14202/vetworld.2020.530-537

**Published:** 2020-03-21

**Authors:** Wurlina Wurlina, Mas’ud Hariadi, Erma Safitri, Suherni Susilowati, Dewa Ketut Meles

**Affiliations:** 1 Department of Veterinary Reproduction, Faculty of Veterinary Medicine, Universitas Airlangga, Surabaya 60115, Indonesia; 2 Department of Veterinary Pharmacology, Faculty of Veterinary Medicine, Universitas Airlangga, Surabaya 60115, Indonesia

**Keywords:** crude tannins, intact plasma membrane, motility, spermatozoa, viability

## Abstract

**Aim:**

The aim of this study was to know crude guava leaf tannins effect on motility, viability, and intact plasma membrane of stored spermatozoa of Etawa crossbred goats.

**Materials and Methods:**

Macroscopic assessment of normal Etawa crossbred semen was followed by dilution with a glucose solution at a 1:10 ratio to increase volume. The diluted semen was treated by adding crude guava leaf tannins into 1 ml of the semen glucose diluent, and five treatments were obtained, namely, control group (C), with no added tannins; treatment Group 1 (T1), with 3%; treatment Group 2 (T2), with 6% tannins; treatment Group 3 (T3), with 12% tannins; and treatment Group 4 (T4), with 24% tannins. Each treatment used five replications. Then, microscopic analysis of the treated and control semen was carried out after 15 days of storage at 4-5°C temperature. The parameters observed were motility, pH, viability, abnormality, and intact spermatozoa plasma membrane.

**Results:**

The spermatozoa motility in Group C was the highest (76.60±1.47). The motility in Group T1 did not differ from that in Group C, but was different and higher than that in Groups T2, T3, and T4. The pH of Group C tended to be acidic after 15 days of storage (4.78±0.01) as compared to the initial pH of fresh semen (6.76±0.12). The pH in Group C did not differ from that in the Groups T1 and T2, but differed from that in the T3 and T4 groups; the pH in the T3 and T4 groups was similar. The viability of spermatozoa in the T1 group was higher than that in all treatments (64.60±2.76); the lowest values were found in Group C (28.94±1.02). Group C had the lowest number of normal spermatozoa, with a mean of 72.58±3.48. The total number of abnormalities in the T2 group did not differ from those in the T3 group, and abnormalities in the T4 group did not differ from those in Group C, which exhibited the highest abnormalities in the head, neck, and tail. The most significant decrease was observed in the intact plasma membrane of spermatozoa on addition of 12% and 24% crude guava leaf tannin in glucose diluents.

**Conclusion:**

The addition of 3% crude guava leaf tannin to crossbred Etawa goat semen diluted with glucose diluent and stored for 15 days at 4-5°C resulted in a significant effect on spermatozoa motility, viability, and intact plasma membrane, whereas the administration of 24% crude guava leaf tannin resulted in low live percentage of spermatozoa.

## Introduction

The productivity of local Indonesian goats is still relatively low compared to that of goats originating from subtropical regions, as the weight of goats at the age of 1 year only reaches around 14-17 kg. This is due to the nonoptimal breeding efforts carried out in goat livestock. However, local goats have the advantage of adaptability and reproductive efficiency, for example, the number of kiddings can reach up to four kiddings per births and 3 times of births per 2 years [1]. Artificial insemination (AI) technology has long been developed in Indonesia, especially in beef cattle and dairy cows, with good results, but its use is still very limited in small livestock such as sheep and goats. The success of AI in goats ranges from 33% to 73%. One of the supporting factors required for optimizing AI programs in goats is the availability of frozen semen that meets the official standards. At present, it is challenging to acquire frozen goat semen that meets the standards suitable for use in AI programs [2].

The use of fresh semen in the AI program is an alternative to the difficulty of procuring frozen semen that meets the official standards. The pregnancy success rates in AI programs in goats using liquid semen are 62.5-73.1% at spermatozoa concentrations of 60-120 million/0.5 ml of semen [3]. The process of storing liquid goat semen is generally carried out at temperatures of 4-5°C to avoid the negative effects of storage; however, the problem is that the composition and characteristics of the glucose diluents needed for storage of liquid semen in goats have not been widely reported [4,5].

To prevent the toxic effects of egg yolk diluent on goat spermatozoa, a more suitable diluent material is needed to improve the viability and integrity of the goat spermatozoa membrane, considering that goats are economically important livestock that is widely maintained by the community because of their easily adaptable nature and easy maintenance. Based on the problem of the toxicity of egg yolk thinners to goat semen, we proposed to improve the motility, viability, and integrity of spermatozoa in the crossbred Etawa goat semen by adding crude tannins [6]. Crude tannins are expected to form complex bonds, with both proteins and polysaccharides. In addition, tannins are also able to bind to complex proteins or proteins bound to calcium (Ca), Magnesium (Mg), Sodium (N)a, and Potassium (K) ions, carbohydrates, and fats [7,8]. The binding of proteins and ions in the spermatozoon membrane causes it to retain available energy until it undergoes maturity in the female reproductive tract so that fertilization can occur [9,10]. Guava leaves contain flavonoids, especially quercetin, saponins, alkaloids, pectin or water-soluble fibers, tannins containing phenols, and essential oils that can be used in antibacterial treatments [11]. Tannins in guava leaves are known as psiditannins, a group of amorphous organic substances with acidic pH, that are able to precipitate alkaloids and glycosides. Besides, tannins are also antibacterial, neutralizing, or absorbent, and astringent compounds. Crude guava leaf contains 9% tannins and 0.4% essential oils that are greenish containing eugenol, 6% fat oil, 3% dammar, and 3% mineral salts [12].

Our research is expected to increase the success of AI in local goats using liquid semen from the crossbred Etawa males to improve genetic and population quality. We added crude guava leaf tannins to the crossbred Etawa semen before it was frozen to preserve the motility, viability, and intact plasma membrane of spermatozoa until day 15 of storage. After that, the semen was evaluated macroscopically for volume, color, consistency, and pH, and microscopically for mass movement, spermatozoa concentration, and motility. The future final purpose was to determine whether the semen was suitable for freezing after added crude guava leaf tannins. The ejaculate volume of sheep/goat semen ranges from 0.8 to 1.2 ml, with a pH of 5.9-7.3, spermatozoa concentrations of 2000-3000 million/ml with 60-80% motility, with 80-95% normal, and 8-10% abnormal spermatozoa [1].

## Materials and Methods

### Ethical approval

Animal handling was carried out with ethical approval by the Animal Care and Ethical Clearance Committee of Faculty of Veterinary Medicine, Universitas Airlangga, and conforms with the National Research Council’s guidelines through the ethical seminar.

### The procedure of extracting guava leaf tannins

The guava leaf tannin extraction process was as follows: A total 1000 g of guava leaves were dried in a confined space at room temperature and in indirect sunlight. After that, the dry matter was weighed to determine the weight loss after the drying process, and then the leaves were ground into a fine powder with a blender and sifted through a filter of sieve size 00.

A total of 100 g of guava leaf samples were placed in a Soxhlet apparatus with 600 ml of n-hexane for 6 h to obtain the filtrate and residue. Then, the residue was refluxed with 400 ml of methanol (MeOH):dichoromethane (CH_2_Cl_2_) (2:1) for 30 min, with three replications to obtain filtrate and residue. Subsequently, the filtrate was concentrated so that a crude extract was formed. Afterward, reflux was carried out with 200 ml of ethyl acetate (CHCl_2_:crude extract; 1:1) for 30 min, with seven repetitions to obtain filtrate and sediments. The precipitate was dissolved with 100 ml MeOH: CH_2_Cl_2_ (2:1) and concentrated to 40 ml. Furthermore, it is dripped on 100 ml acetone to obtain filtrate and sediment. The precipitate was placed in a 400°C oven to obtain guava leaf extract. Furthermore, we used spectrophotometry (Evolution™ 201/220 Ultraviolet-visible Spectrophotometers, Catalog No 912A0883, Thermo Fisher Scientific, USA) to determine the tannin and phenol content of guava leaf powder [13]. A total of 1.5 kg of guava leaf flour produced guava leaf extract of 17.825 g with a tannin content of 2.41% and phenolic content of 20.80%.

### Semen collection of Etawa crossbred goats

This study used a 2-year-old Peranakan Etawa male from whom semen was collected alternately at each shelter in Teaching Farm, Gresik, Indonesia. Semen collection of Etawa crossbred goat was carried out in the morning at 08.00 h West Indonesia Time with five repetitions twice every week, following the preparation stages of introduction of an artificial vagina, preparation of the male, and holding of semen.

Macroscopic and microscopic semen evaluation was carried out immediately after collection. The macroscopic examination included testing the volume, consistency, odor, color, and acidity, while microscopic examination included checking mass and individual sperm movements and sperm concentration. A good macroscopic value of goat semen is an average of 0.8-2 ml of relatively thick consistency, a specific or normal characteristic milky odor, yellowish-white color, with a 6.2-6.8 pH. Microscopic assessment of goat semen is good when mass movement with waves is ++ (positive 2), individual movements are 70%/3 with movement +++ (positive 3), and spermatozoa concentrations are approximately 1000×10^6^ spermatozoa/mL.

### Treatment of Etawa crossbred semen

After the macroscopic assessment, normal goat semen is diluted with a glucose solution to increase the volume by a ratio of 1:10 [14]. The diluted semen was treated by adding crude guava leaf tannin at concentrations of 3%, 6%, 12%, and 24% into 3 ml of the semen glucose diluent, to obtain five treatments, i.e., the control group (C), with no added tannins; treatment Group 1 (T1), with 3% tannins in the semen glucose diluent; treatment Group 2 (T2), with 6% tannins in semen glucose diluent; treatment Group 3 (T3), with 12% tannins in the semen glucose diluent; and treatment Group 4 (T4) with 24% tannins in the semen glucose diluent.

This study was repeated 5 times and microscopic analysis was carried out after 15 days of storage at 4-5°C. The parameters observed were pH, motility, viability, abnormality, and intact plasma membranes of the spermatozoa.

### Analysis of spermatozoa motility

Analysis of spermatozoa motility was carried out by dropping one drop of semen on a glass slide and close with a cover glass. The same number of droplets was tested for each inspection. Spermatozoa motility was determined from that of 100 spermatozoa in one field of view. The spermatozoa motility was assessed according to the following criteria:

a. Spermatozoa with good motility, i.e., fast, straight-forward movement, agile, and active (%)

b. Spermatozoa with poor motility, i.e., any motion other than that in spermatozoa with good motility (%)

c. Non motile spermatozoa (%).

### pH analysis (degree of acidity)

A pH meter or litmus bag was used for the analysis of the pH of semen. Normal goat semen has a pH between 6 and 7. The better is the quality of the semen, the more it tends to be acidic; this is because the more active spermatozoa in good quality semen move and produce more lactic acid, resulting in the lowering of the pH.

### Spermatozoa viability analysis

**Figure-1:**
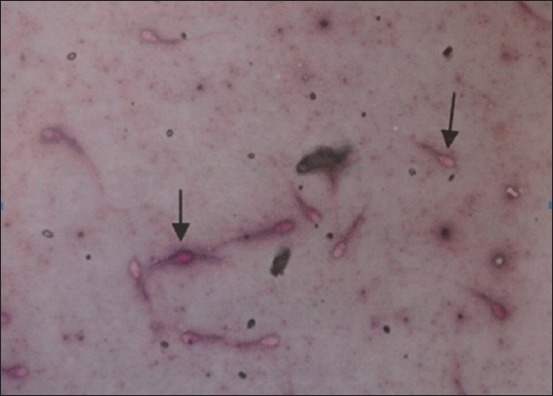
Viability of spermatozoa live spermatozoa: The head does not absorb color dead spermatozoa: The head absorbs color.

Spermatozoa viability analysis was carried out by dripping one drop of semen onto a glass plate and then being stained with an eosin-nigrosin coloring. The same number of droplets was examined for each inspection. Spermatozoa viability was determined from 100 spermatozoa in one field of view. Spermatozoa viability was assessed according to the following criteria (Figure-1):

a. The heads of live spermatozoa do not absorb color (%)

b. The heads of dead spermatozoa absorb color (%).

### Analysis of spermatozoa abnormalities

Spermatozoa abnormality analysis was carried out by dripping one drop of semen on a glass slide and staining with an eosin-nigrosin dye. The same number of droplets was examined for each inspection. Spermatozoa abnormalities were determined from 100 spermatozoa in one field of view. Assessment of spermatozoa abnormalities was based on the following criteria:

a. Abnormalities in the head of the spermatozoa (%)

b. Abnormalities in the neck of the spermatozoa (%)

c. Abnormalities in the tail of the spermatozoa (%).

### Whole plasma membrane analysis

Analysis of spermatozoan intact plasma membranes was carried out using the hypoosmotic swelling test (HOS) to assess the integrity and function of spermatozoan membranes. This test uses a chemical (2.7 g fructose and 1.47 natrium sitrat in aquadest 100 mL) that increases the osmotic pressure outside the spermatozoa cells in comparison to that inside the cells, which causes the plasma cells of the spermatozoa to swell from head to tail, and the spermatozoa tails become abnormal.

**Figure-2:**
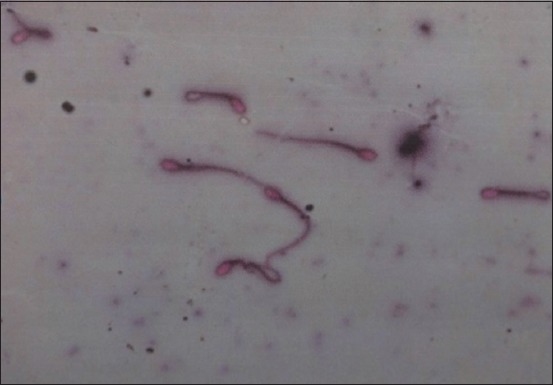
Intact plasma membrane of spermatozoa Intact plasma membrane: Circular spermatozoa tail damaged plasma membrane: Straight spermatozoa tail.

The tails of the spermatozoa in the hypotonic medium can form a spiral. This is due to the interruption of contraction and relaxation in the tail of spermatozoa due to the entry of ions which have low molecular weight. The integrity of spermatozoa cell membrane can be disturbed by adding the HOS solution and then cause spermatozoa cell swelling and membrane damage that forms a straight tail (Figure-2) [15]. Assessment of intact plasma spermatozoa was based on the following criteria:

a. Spermatozoa with damaged membranes have straight tails (%);

b. Good membrane spermatozoa exhibit bent and circular tails (%).

### Statistical analysis

The data from spermatozoa characteristics, such as volume, pH, color, consistency, motility (mass movement), and concentration, were analyzed descriptively; whereas spermatozoa quality data, such as motility (individual movements), pH viability, abnormality, and intact plasma membrane, in semen stored for 15 days were analyzed using one-way analysis of variance. If there were real differences, we proceeded with the Tukey W-Procedure test.

## Results

### Fresh semen quality evaluation

On macroscopic and microscopic examination of normal semen from 5 male goats in this study, it was found that the average volume of semen per ejaculate was 1.39±0.03 ml. The acidity (pH) of fresh goat semen obtained in this study was 6.76±0.12. The consistency of fresh goat semen obtained in this study was thick/thick, and the color of fresh goat semen was like that of creamy milk. The smell of fresh goat semen has a typical milky smell (specific). The mass movement was ++; the individual movement was 80/3. Spermatozoa concentration was (328.8±80.74)×10^7^ spermatozoa/ml, which is normal goat semen concentration. Fresh semen samples collected from Etawa crossbred male goats were analyzed to determine the average macroscopic and microscopic values and are presented in Table-1.

**Table-1:** Average of macroscopic and microscopic semen values.

Characteristic	Average
Volume	1.39±0.03 ml
pH	6.76±0.12
Color	Creamy milk
Consistency	Thick
Motility	+++
Concentration	(321.83±16.00)×10^7^ ml semen

### Spermatozoa motility

The average percentage of spermatozoa motility after adding crude guava leaf tannins and storing for 15 days is shown in Table-2. Group C exhibited the highest motility values (76.60 ± 1.47). The motility in the T1 group did not differ from that in Group C, but was different and higher than that in the T2, T3, and T4 groups, which shows that there were still spermatozoa that were moving even though it was not progressive movement.

**Table-2:** Average of percentage and standard deviation of spermatozoa motility for 15 days.

Treatment	Average value of motility (%)
C	76.60^a^±1.47
T1 (crude tannin 3%)	74.44^a^±1.39
T2 (crude tannin 6%)	63.72^b^±1.94
T3 (crude tannin 12%)	58.32^c^±7.45
T4 (crude tannin 24%)	56.68^c^±5.60

Different superscripts indicate significant differences (p<0.05)

Motility in the T2 group also showed higher mean motility than that in the T3 and T4 groups. The T3 group had a lower mean motility value than that in the T1 and T2 groups, and similar was the case with the value of T4, which had the lowest mean motility value.

### pH or degree of acidity of the semen

The average pH of semen after the addition of crude guava tannins and storage for 15 days is shown in Table-3. The pH of Group C tends to be acidic at 4.78±0.01 after 15 days as compared to the initial pH of fresh semen, which was 6.76±0.12. The results of the study of pH in Group C were similar to those in the T1 and T2 groups but were different from those in the T3 and T4 groups; however, there was no difference between the pH of the T3 and T4 groups.

**Table-3:** Average of pH and standard deviation of spermatozoa for 15 days.

Treatment	Average value of pH
C	4.78^a^±0.01
T1 (crude tannin 3%)	4.79^a^±0.12
T2 (crude tannin 6%)	4.78^a^±0.02
T3 (crude tannin 12%)	4.67^b^±0.08
T4 (crude tannin 24%)	4.64^b^±0.10

Different superscripts indicate significant differences (p<0.05)

### Viability of spermatozoa

The average percentage of spermatozoa viability stored for 15 days after the administration of crude guava tannins is shown in Table-4. The viability of spermatozoa in the T1 group (64.60±2.76) was higher than that in all treatments, and Group C had the lowest value of 28.94±1.02.

**Table-4:** Average of percentage and standard deviation of live spermatozoa for 15 days.

Treatment	Live spermatozoa (%)
C	28.94^e^±1.02
T1 (crude tannin 3%)	64.60^a^±2.76
T2 (crude tannin 6%)	57.63^b^±2.70
T3 (crude tannin 12%)	51.47^bc^±2.18
T4 (crude tannin 24%)	46.84^d^±1.27

Different superscripts indicate significant differences (p<0.05)

### Spermatozoa abnormalities

The average percentage of spermatozoan abnormalities after the addition of crude guava tannins and storage for 15 days is in Table-5. Group C had the lowest normal spermatozoa, which was 72.58%±3.48%. In the T1 group, the percentage of normal spermatozoa was higher than that in Group C and the T2, T3, and T4 groups. The mean percentages abnormalities of the T2 group did not differ from the T3 group, whereas those in the T4 group did not differ from those in Group C, which had the highest abnormalities in the head, neck, and tail of the spermatozoa.

**Table-5:** Average of percentage and standard deviation of spermatozoa abnormalities for 15 days.

Treatment	Spermatozoa abnormalities (%)				

	Normal spermatozoa	Head abnormality	Neck abnormality	Tail abnormality	Total abnormalities (head+neck+tail)
C	72.58^c^±3.48	10.35^b^±0.45	8.63^a^±0.38	9.49^a^±0.64	28.47^a^±1.47
T1 (crude tannin 3%)	86.44^a^±3.32	5.68^d^±0.46	4.83^c^±0.45	4.78^c^±0.28	15.29^c^±1.19
T2 (crude tannin 6%)	78.79^b^±1.51	8.51^c^±0.80	7.49^b^±0.47	6.99^b^±0.28	22.99^b^±1.55
T3 (crude tannin 12%)	77.08^b^±1.21	10.75^b^±0.85	7.44^b^±0.42	5.31^c^±0.35	23.50^b^±1.62
T4 (crude tannin 24%)	73.80^c^±3.38	13.38^a^±0.50	7.63^b^±0.49	6.69^a^±0.69	27.70^a^±1.68

Different superscripts at the same column indicate significant differences (p≤0.05)

### Whole plasma membrane spermatozoa

The average percentage of intact plasma mem­brane (IPM) of spermatozoa after the addition of crude guava tannins and storage for 15 days is shown in Table-6. In this study, we observed a decrease in the intact plasma membrane of the spermatozoa on addition of guava leaf crude tannin; addition of 12% and 24% tannins in glucose diluents resulted in the lowest number for intact spermatozoa plasma membrane. It could be concluded that the higher is the concentration of crude tannin leaf guava; the lower is the percentage of intact plasma membrane spermatozoa. It is possible that the addition of crude guava leaf tannin at a concentration of 12% and 24% was toxic, and thus, it could not maintain the integrity of the spermatozoa plasma membrane.

**Table-6:** Average of percentage and standard deviation of the spermatozoa plasma membrane for 15 days.

Treatment	Average value of membrane integrity (%)
C	76.15^a^±1.08
T1 (crude tannin 3%)	74.55^a^±0.82
T2 (crude tannin 6%)	63.52^b^±1.08
T3 (crude tannin 12%)	58.33^c^±1.27
T4 (crude tannin 24%)	57.17^c^±1.69

Different superscripts indicate significant differences (p<0.05)

## Discussion

Macroscopic and microscopic examination of normal fresh semen is very important to assess the suitability of the semen for research. The normal volume of goat semen per ejaculate is 0.8-1.2 ml on average. The acidity (pH) of the semen is between 5.9 and 7.3. The normal consistency of goat semen is moderate to thick/thick. The standard color of goat semen is milky beige. The standard odor of goat semen is specific or normal. Fresh semen that is processed into frozen semen has a minimum mass movement value of ++ (2) and individual movements with at least 70/3 motility of active spermatozoa moving forward (progressive). Calculation of spermatozoa concentrations was carried out with a spectrophotometer, and the values were found to be 1500-3000×10^6^ spermatozoa/ml [1,9].

In this study, it was found that the average volume of semen per ejaculate was 1.39±0.03 ml. The difference in the volume of semen produced from a male varies. The volume of semen and the number of spermatozoa produced from each ejaculation are influenced by species, age, season, environment, frequency of ejaculation, feeding conditions, and health [1]. The acidity (pH) of fresh goat semen obtained in this study was 6.76±0.12. The pH of semen tends to be more acidic because many spermatozoa are still actively moving and producing lactic acid. The acidic conditions for a prolonged period can be toxic to spermatozoa; thus, many spermatozoa were found dead in semen with low pH. The examination of fresh semen must be carried out quickly so that the percentage of spermatozoa that live is above 80%, which is desirable for research.

The consistency of fresh goat semen obtained in this study was thick/thick, and the color of fresh goat semen was like that of creamy milk. The color of semen is a reflection of semen thickness and concentration of spermatozoa; the higher is the concentration of spermatozoa in semen, the thicker is the consistency, and the more concentrated is the color [6]. The smell of fresh goat semen was typical (specific). The mass spermatozoa movement was ++, and the individual movement was 80/3. Spermatozoa concentration was (328.8±80.74)×10^7^ spermatozoa/ml, and this was the normal goat semen concentration. The concentration of semen when ejaculated was highly dependent on the process of spermatogenesis. Spermatogenesis is influenced by the follicle-stimulating hormone, which plays a role in stimulating the germinative cells of the seminiferous tubules and testes [1]. The fresh semen samples from Etawa crossbred male goats with average macroscopic and microscopic values are presented in Table-1.

The results showed that the quality and quantity of fresh spermatozoa were appropriate for processing with the diluter. The concentration of semen was influenced by the method and frequency of storage [16]. The collection of semen with an artificial vagina produced a higher concentration than the electro-ejaculator method and increasing the frequency of ejaculation reduced the concentration of spermatozoa.

The average percentage of spermatozoa motility was calculated after the addition of crude guava tannins and storage for 15 days. The spermatozoa motility was highest in Group C (76.60±1.47). This means that spermatozoa without crude guava leaf tannins had a progressive level of movement due to the presence of unhindered metabolic activity in the spermatozoa plasma membrane; however, the spermatozoa would die quickly. The energy used for metabolism in the spermatozoa is high, resulting in dead or immotile spermatozoa as they run out of energy. The motility of the spermatozoa in the T1 group did not differ from that in Group C, due to the formation of tannin bonds with the spermatozoa membrane which limited the movement of spermatozoa; however, it differed and was higher than the motility in the Groups T2, T3, and T4, which shows there were still spermatozoa that were moving even though it was not progressive.

Motility in the T2 group also showed a higher mean motility value than that in the T3 and T4 groups. This was due to the presence of tannin bonds with the spermatozoa membrane. The lower motility values of the spermatozoa in the T4 group were due to the higher level of crude guava leaf tannin concentration in this group as compared to that in Groups T1, T2, and T3 – as the higher the crude tannin added, the higher is the phenol content which is toxic to the spermatozoa resulting in many spermatozoa deaths. The increasing tannin levels can also inhibit the movement of spermatozoa because tannins can bind to proteins in spermatozoa plasma membrane or can bound to Ca, Mg, Na, and K ions, carbohydrates, and fats in seminal plasma. Seminal plasma is a factor that affects motility.

This energy from protein, mineral, carbohydrates, and fat will be used by spermatozoa traveling to the ampulla so that premature capacitation does not occur and that the possibility of fertilization is higher. Capacitation is the change experienced by a spermatozoon for gaining power for fertilization and involves enzymes found in the head of spermatozoon [17]. Spermatozoa capacitance is indicated by an increase in tyrosine and osteopontin which are the main proteins in membrane fibers [18,19].

Capacitation plays an important role in the acrosomal changes needed by spermatozoa for egg penetration. After spermatozoa capacitation, there is hyperactive motility in mammalian spermatozoa [20]. In the beginning, capacitation involved the rearrangement of fat in the spermatozoa plasma membrane, producing ionic changes in the spermatozoa membrane alteration, and an increase in tyrosine phosphorylation of proteins that induce acrosome reactions and hyperactivation [10].

The average pH of semen after the addition of crude guava tannins and storage for 15 days, as shown in Table-3, depicts the pH of Group C tended to be acidic, at 4.78±0.01, as compared to the primary pH of fresh semen which was 6.76±0.12. One of the factors influencing the jump in pH was the effect of the duration of spermatozoa storage on their metabolism, i.e., the longer the spermatozoa was stored, the lower pH would tend to decline because of the gradual rise of spermatozoa metabolism. This leads to an increase in lactic acid formation in massive amounts in the spermatozoa. The dip in pH toward acidity is brought about by a decrease in the metabolism rate (MR) in spermatozoa, whereas at neutral pH, the MR tends to increase. Metabolism speed is highly dependent on energy usage, and the anaerobic conditions during storage affect the drop in pH and lactic acid accumulation [4,17].

The results of the study of pH in Group C did not differ from those in the T1 and T2 groups, whereas the results between T3 and T4 groups were similar. The addition of higher concentrations of raw guava leaf tannins resulted in more acidic pH. This was because guava leaf tannins contain phenolic compounds with acidic properties that can release H+ ions from their hydroxyl groups. The removal of these ions makes the phenolate anion fenoksida C_6_H_5_O soluble [4,21]. Phenolic compounds are a part of poisonous compounds in plants that can have an impact when used at high levels. The Ca^2+^ ions present in the spermatozoa plasma membrane can be bound by polyphenols so that ion breakdown or exchange can occur [10,17].

The average percentage of spermatozoa viability after the administration of crude guava tannins and storage for 15 days in the T1 group was higher than that in all treatments at 64.60±2.76 and that in Group C was the lowest at 28.94±1.02. The addition of raw guava leaf tannin gives the spermatozoa life force in all treatments. Motility, pH, and abnormalities are factors that can affect the viability of spermatozoa, where pH is very influential in spermatozoa activity. If spermatozoa are at a neutral pH (7.0), it increases the average MR and decreases metabolism when it becomes alkaline or acidic. pH can extend the viability of spermatozoa by reducing its activity [4,22]. The level of abnormal spermatozoa is an important factor because normal spermatozoa have longer viability compared to that of abnormal spermatozoa, and normal spermatozoa have the ability to fertilize before losing their motility [23]. Thus, the addition of crude guava leaf tannins at 3% concentration in liquid spermatozoa from Etawa crossbred goats has a significant effect on their viability so that spermatozoa have low activity, which results in spermatozoa reserving energy for fertilization which can be made possible progressively while in the female reproductive channel because tannins can be released by themselves at low pH [18]. Spermatozoa use fructose present in seminal plasma under anaerobic conditions (without oxygen) during storage. Lactic acid produced from fructose metabolism can be stored again and can be used as energy during aerobic conditions [22,24].

Morphological abnormalities of spermatozoa can originate from primary damage during spermatozoa formation (spermatogenesis) and during the maturation process in the epididymis and from secondary damage arising from the collection and evaluation of sperm [16]. The degree of abnormality is an important factor in AI because only normal and intact spermatozoa have a higher chance of successful fertilization.

The values for the average percentage of spermatozoan abnormalities after the addition of crude guava tannins and storage for 15 days are shown in Table-5. Group C had the lowest normal spermatozoa at 72.58±3.48. This could be caused by the absence of a protective covering on the spermatozoa so that normal spermatozoa have the lowest mean value as compared to spermatozoa with crude guava leaf tannins. In the T1 group, the number of normal spermatozoa was higher than that in Group C and the T2, T3, and T4 groups, due to the tannin as a protective layer of plasma membrane. The head, body, and tail also had relatively small abnormalities in Group T1.

The average abnormalities of the T2 group were the same as those in T3; whereas the abnormalities in the T4 group did not differ from those in Group C, which had the highest abnormalities in the head, neck, and tail of the spermatozoa. This is due to the increased concentration of guava leaf tannins in T4 (24%), which results in lower or fewer normal spermatozoa due to higher phenolic content.

Sperm with spermatozoan abnormalities of more than 20% cannot be used for fertilization [23]. Spermatozoa head damage in the post-nuclear cap originating from the nuclear membrane is a sign of primary damage originating during spermatogenesis. Acrosomal damage to the head of spermatozoa, usually found in Friesian cows, affects spermatozoa during ejaculation and makes cattle sterile. Spermatozoa neck damage can be caused due to hot temperatures or in a state of stress, and the neck can experience damage that causes the separation of the head and tail[10]. Separation of the head and tail of spermatozoa occurs within the head of the epididymis [25]. More than 60% of the short head and tail are cutoff at 2-3 μm. Pseudo droplets are characteristic of spermatozoa due to the influence of a twisted or extended neck. Abnormalities in the neck or middle part of spermatozoa at 15-50% will cause spermatozoa to die [23].

The integrity of the plasma membrane is the integrity of spermatozoa and is very instrumental in the process of fertilization for the success of AI. The function of the membrane is to regulate the entry and exit of nutrients, ions needed in the process of metabolism, and to maintain intra- and extra-cellular electrolytes [19]. Intact plasma spermatozoan membranes are characterized by swelling and coiling at the tail end of the spermatozoa, short, and thick tails, or swelling in part or all parts formed by the tail of spermatozoa; whereas damaged spermatozoa are marked with a straight tail [15]. The form and characteristics of spermatozoa damaged due to lipid peroxidation are decreased motility, intracellular enzyme damage, and damage to the structure of the plasma membrane [26]. The average percentage of the IPM of spermatozoa after the addition of crude guava tannins and storage for 15 days is shown in Table-6.

Intact plasma membrane is needed by spermatozoa because damage to the plasma membrane affects the metabolic process and is related to motility and survival of spermatozoa. Cell metabolism will take place well if the spermatozoa plasma membrane is intact so that it can regulate the entry and exit of electrolytes needed in the metabolic process [27,28]. During spermatozoa maturation in the epididymis, there is also a change in the composition of the components making up the spermatozoa plasma membrane. Spermatozoa plasma membrane will lose some cholesterol, resulting in an increase between unsaturated fatty acids and cholesterol [29,30].

In this study, we found the greatest decrease in the intact plasma membrane of spermatozoa on the addition of guava leaf crude tannin at concentrations of 12% and 24% in glucose diluents. It could be concluded that the higher the concentration of crude tannin leaf guava, the lower would be the percentage of intact plasma membrane spermatozoa. The addition of crude guava leaf tannin at concentrations of 12% and 24% was toxic, so it could not maintain the integrity of the spermatozoa plasma membrane. A decrease in the quality of spermatozoa during storage, the percentage of progressive motility, and the integrity of the plasma membrane due to the number of spermatozoa that die and become toxic to other spermatozoa that are still alive, lead to a general decrease in the quality of spermatozoa [21,30]. Toxic substances derived from dead spermatozoa or from raw guava leaf tannins or from retailers (semen glucose diluent) that have been oxidized due to storage, can cause high levels of free radicals that can damage the integrity of the spermatozoa plasma membrane [31]. If the spermatozoon’s plasma membrane is damaged, it will lead to the death of spermatozoa.

## Conclusion

The addition of crude guava leaf tannins at a concentration of 3% can increase motility, viability, and preserve intact plasma membrane in Etawa crossbred goat semen diluted with glucose and stored at 4-5°C, whereas the administration of crude guava leaf tannins at concentrations of 24% will result in a low percentage of live spermatozoa.

## Authors’ Contributions

WW: Research project leader, research, and ethical clearance preparation, conducted main researchand drafted the manuscript. MH: Evaluation of Etawa crossbreed semen (micros and macros), proofread for manuscript. ES: Analyzed statistical data, proofreading, andprepared the final version of the manuscript. SS: Semen collection, evaluation, treatment of Etawa crossbreed semen. DKM: Designed study, prepared guava leaf tannin extraction and observation of IHC. All authors read and approved the final manuscript.
